# Prediction of cardiac index by body surface temperatures, ScvO_2_, central venous-arterial CO_2 _difference and lactate

**DOI:** 10.1186/cc9454

**Published:** 2011-03-11

**Authors:** W Huber, B Haase, B Saugel, V Phillip, C Schultheiss, J Hoellthaler, R Schmid

**Affiliations:** 1 Klinikum Rechts der Isar der Technischen Universität München, Germany

## Introduction

Monitoring of the cardiac index (CI) is a cornerstone of intensive care. Nevertheless, most of the techniques based on indicator dilution and/or pulse contour analysis require central venous and/or arterial catheters. Several surrogate markers have been suggested to estimate CI including ScvO_2_, central venous-arterial CO_2 _difference (CVACO_2_D) as well as body surface temperatures and their differences to body core temperature (BCT). It was the aim of our prospective study to evaluate the predictive capabilities of CVACO_2_D, ScvO_2_, surface temperatures and lactate regarding CI.

## Methods

In 53 patients (33 male; 20 female) with PiCCO monitoring, 106 datasets including surface temperatures of great toe, finger pad, forearm and forehead using an infrared noncontact thermometer (Thermofocus; Tecnimed) as well as lactate, ScvO_2_, CVACO_2_D and pulse pressure (PP) were measured immediately before PiCCO thermodilution providing CI and SVRI. Statistics: SPSS 18.0.

## Results

Patients: 17/53 (32%) ARDS; 14/53 (26%) liver cirrhosis; 13/53 (25%) sepsis; 4/53 (8%) cardiogenic shock; 5/53(9%) various aetiologies. Thermodilution-derived CI significantly correlated to the temperatures of the forearm (*r *= 0.465; *P *< 0.001), great toe (*r *= 0.454; *P *< 0.001), finger pad (*r *= 0.447; *P *< 0.001) and forehead (*r *= 0.392; *P *< 0.001) as well as to ScvO_2 _(*r *= 0.355; *P *< 0.001), SCVACO_2_D (*r *= -0.244; *P *= 0.011) and pulse pressure (*r *= 0.226; *P *= 0.019), but not to lactate (*r *= -0.067; *P *= 0.496). ROC analysis regarding the critical threshold of CI <2.5 l/minute*sqm demonstrated the highest predictive capabilities for the differences (BCT - T-forearm) (ROC-AUC 0.835; *P *= 0.002; cut-off 4.6°; sensitivity 89%; specificity 71%) and (BCT - T-finger pad) (ROC-AUC 0.757; *P *= 0.017) as well as ScvO_2 _(ROC-AUC 0.744; *P *= 0.024). SCVACO_2_D (ROC-AUC 0.706; *P *= 0.056) and lactate (ROC-AUC 0.539; *P *= 0.718) were not predictive. Multiple regression analysis (*R *= 0.725) demonstrated that age (*P *< 0.001), PP (*P *< 0.001), T-forearm (*P *= 0.024) and the difference (BCT - T-toe; *P *= 0.035) were independently associated with CI.

## Conclusions

Body surface temperatures and their differences to BCT are useful to estimate CI. The difference (BCT - T-forearm) provided the largest ROC-AUC (0.835; *P *= 0.002) regarding CI <2.5 l/minute*sqm. SCVACO_2_D does not provide information in addition to body surface temperatures and ScvO_2_.

